# Resistant starch, microbiome, and precision modulation

**DOI:** 10.1080/19490976.2021.1926842

**Published:** 2021-07-18

**Authors:** Peter A. Dobranowski, Alain Stintzi

**Affiliations:** a Department of Biochemistry, Microbiology and Immunology, University of Ottawa, Ottawa, Ontario, Canada; b Ottawa Institute of Systems Biology, University of Ottawa, Ottawa, Ontario, Canada

**Keywords:** Resistant starch, microbiome, personalized therapies, personalized medicine, precision medicine, clinical trials

## Abstract

Resistant starch, microbiome, and precision modulation. Mounting evidence has positioned the gut microbiome as a nexus of health. Modulating its phylogenetic composition and function has become an attractive therapeutic prospect. Resistant starches (granular amylase-resistant α-glycans) are available as physicochemically and morphologically distinguishable products. Attempts to leverage resistant starch as microbiome-modifying interventions in clinical studies have yielded remarkable inter-individual variation. Consequently, their utility as a potential therapy likely depends predominantly on the selected resistant starch and the subject’s baseline microbiome. The purpose of this review is to detail i) the heterogeneity of resistant starches, ii) how resistant starch is sequentially degraded and fermented by specialized gut microbes, and iii) how resistant starch interventions yield variable effects on the gut microbiome.

## Introduction

Dietary fibers promote human health, in large part through the gut microbiome. A prebiotic is defined as “a substrate that is selectively utilized by host microorganisms conferring a health benefit”.^1^ Non-digestible carbohydrates are prebiotics that gut microbes ferment into beneficial metabolites like butyrate.^1^ Butyrate is recognized as a key contributor to host health by maintaining immune homeostasis, gut barrier integrity, and metabolism (reviewed in ref. 2).^2^ Consequently, non-digestible carbohydrates have attracted immense interest as potential adjuvant therapies for many health disorders.

Resistant starch (RS) is among the recent foci of non-digestible carbohydrate therapies. Boosting gut butyrate production has been the objective of several RS intervention studies associated with aging,^3^ insulin resistance,^4^ metabolic syndrome,^5^ kidney disease,^6^ and schizophrenia,^7^ and may be especially relevant for illnesses characterized by dysregulated epithelial integrity and immune function, like inflammatory bowel disease.^8^

Upon reaching the colon, RS acts as a communal resource that is degraded and fermented by a hierarchy of specialized gut microbes. RS selectively feeds upstream keystone species, which produce substrates that are cross-fed upon by butyrogenic bacteria (recently reviewed in ref. 9).^9^ However, the variable effects of RS on the gut microbiome are striking, whereby RS supplementation may increase butyrate production in one person and lower it in another.^10,11^ Such variability underscores the need for precision nutrition to confer health benefits. Predicting the most optimal RS for individual demands understanding the complex structures of RS granules, the enzymatic machinery required to degrade RS, and the individual baseline microbiome. Hence, the goal of this review is to:

1. Describe the physicochemical and morphological heterogeneity of starch, and explain how these properties confer resistance to host and bacterial hydrolysis;
2. Detail the hierarchy of gut bacteria known to degrade and ferment resistant starch; and
3. Highlight the importance of precision resistant starch interventions.

## Section 1: Resistant starch

In humans, digestible starch is susceptible to hydrolysis by salivary and pancreatic α-amylases, which hydrolyze α-1,4-glycosidic bonds.^12^ Starch that reaches the large intestine without being fully digested is termed resistant starch (RS). Resistance depends on several physicochemical features, including physical encasement in non-digestible material (type 1), native supramolecular structure and morphology (type 2), retrogradation via hydrothermal-cycling (type 3), chemical modifications (predominantly ester cross-linking) (type 4), and amylose-lipid complexes formed during cooking (type 5). Types 2, 3, and 4 RS are the primary RS types used in human studies and will be the focus of this review. Extrinsic factors, such as host amylase gene copy number,^13^ oro-cecal transit time,^14^ and amylase inhibition,^15^ further complicate starch bioavailability. Hence, starch digestibility should be considered as a kinetic property (slower to faster) affected by host-specific factors, rather than as a binary trait (resistant or nonresistant).^12^

### Type 2 RS – Native starch

**Figure 1.**
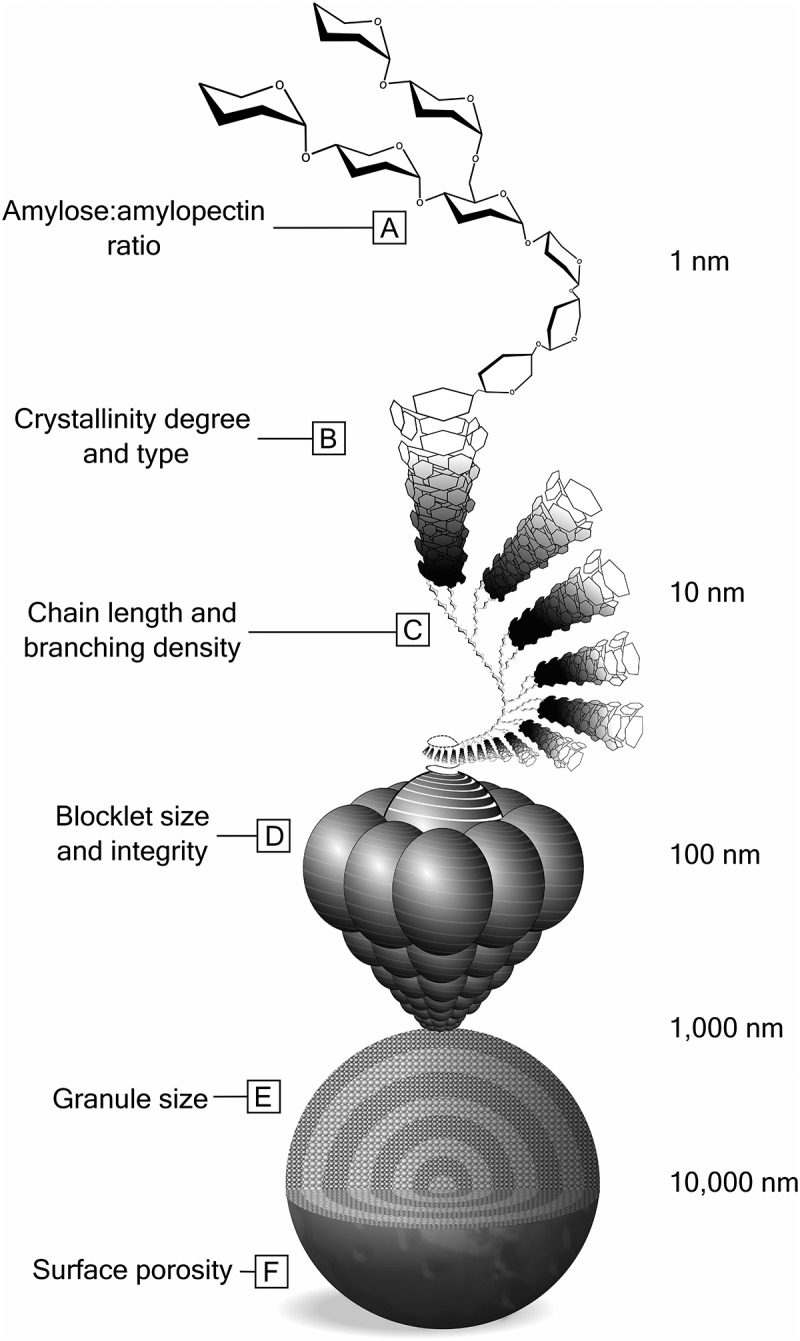
**The hierarchical structure of starch granules**. The physicochemical properties (left-side) of glucose polymers influence the overall morphology and digestibility of the starch granule. The scale of each structural level is indicated on the right-side

Starch is synthesized in the amyloplast and chloroplast organelles of plants, forming mixtures of amylose and amylopectin. These molecules both consist of chains of glucose subunits linked by α-1,4- and α-1,6-glycosidic bonds, but differ in their chain length (i.e. degree of polymerization; DP) and branching (α-1,6 bonds). Amylose possesses a DP below 6,300 glucose subunits, almost entirely (>99.3%) bonded by α-1,4-glycosidic linkages.^16^ Conversely, amylopectin forms much larger molecules (DP up to 26,500) with dense networks of short chains (mean DP 15–18) branching from longer chains (mean DP 48 to 60).^16^ The intra- and intermolecular interactions of amylose and amylopectin impart starch granules with a complex hierarchical structure (Figure 1).

Starch’s supramolecular structure begins with pairs of amylopectin branch chains and amylose chains intertwining to form crystalline double helices. At amylopectin junction points (i.e. α-1,6 bonds), amorphous regions are thermodynamically favored over helices. Helices can either be dense and orthorhombic (A-type) or open and hexagonal (B-type).^17^ Alternating amorphous and crystalline lamellae are thought to form 20–500 nm intermediary structures called “blocklets”^18,19^ with amorphous amylose forming an intermolecular matrix or “glue” between blocklets.^20^ Recent *in silico* modeling proposes that the blocklet architecture follows phyllotaxic rules, whereby interlocking crystalline platelets form ellipsoid fractals.^21^ Depending on the amylopectin branching density and chain lengths, blocklets might be more amorphous (“defective blocklets”) or crystalline (“normal blocklets”).^18,22^ Blocklets arrange into arrays of alternating crystalline and semi-crystalline concentric rings, disrupted by amorphous channels and veins.^19,20,22,23^ Foresti and colleagues elegantly demonstrated that amorphous layers are preferentially degraded by soluble α-amylase, leaving behind a crystalline skeleton.^24^ Overall, blocklet type and arrangement are associated with surface smoothness, porosity, and resistance to hydrolysis.

Across botanical sources, native starch granules vary by size, degree, and type of crystallinity; surface porosity and texture; relative amylose and amylopectin content; and amylopectin branch chain length and density. As a result, starch digestion rates can vary remarkably. For instance, starch granules from tubers tend to be among the most hydrolysis-resistant native starches, possibly because they are larger,^23,25,26^ enriched in B-type crystallites,^25^ possess longer amylopectin branch chains,^25^ and have a smoother surface texture with fewer pores.^23,25–27^ Huang and colleagues showed that smaller, densely packed blocklets form a resilient shell on the surface of potato starch granules, while the interior is composed of larger, loosely packed blocklets.^28^ The surface porosity, crystallinity, and RS content of corn starch granules correlate with amylose content; high-amylose varieties exhibit less porous surfaces, higher proportions of B-type crystallites, longer amylopectin side chains, and higher resistance to hydrolysis than varieties with no amylose (i.e. “waxy” corn starch).^25,29^ Intriguingly, corn starch resistance peaks with an amylose content of 68%,^30^ suggesting that both amylose and amylopectin are required to confer resistance to hydrolysis.

### Type 3 RS – Retrograded starch

Retrogradation occurs when starch undergoes gelatinization followed by a thermodynamically driven reconfiguration of amylose and amylopectin into a new ordered state. First, when starch is heated in the presence of water,^31^ amorphous regions absorb water, the granule swells, and amylose leaches through surface pores.^19^ The hydrogen bonds stabilizing the helices become disrupted, causing amylose and amylopectin to unwind and dissociate.^32^ Upon cooling, new helices are formed, but the degree to which crystallinity increases depends on the amylose content, cooling temperature, and number of repeated cycles. Retrogradation occurs more rapidly for amylose (over the span of minutes to hours) than amylopectin (over days), due to amylopectin’s complex branching networks.^33^ Colder retrogradation conditions promote the formation of B-type crystallites over A-type,^34,35^ and additional cycles of retrogradation are understood to further increase starch crystallinity and resistance to hydrolysis.^27,36^ It is thought that the formation of smaller blocklets during retrogradation is favored in starches with higher amylose content and longer amylopectin chains.^37^ It seems that low surface porosity, enriched B-type crystallinity, and small blocklets formed during retrogradation could confer resistance to hydrolysis analogous to the intrinsic features seen in potato starch.

### Type 4 RS – Chemically modified starch

Chemical treatments that introduce cross-links strengthen starch structural networks and generally increase resistance to hydrolysis. Treatment with sodium trimetaphosphate (STMP) and sodium tripolyphosphate (STPP) creates phosphoester bridges between glucose residues in amylose and amylopectin.^38^ Higher concentrations of STMP and STPP (10–12% w/w) increase the degree of cross-linking and resistance to hydrolysis.^39^ However, lower concentrations (5%) may increase the digestibility of high-amylose corn starch (HACS), likely because the imparted cross-linking is not sufficient to compensate for the gelatinization that occurs during heat treatment.^40^ Cross-linking can also be induced through specific heat and acid treatment, which forms esters between glucose hydroxyl groups.^41^ Cross-linking is restricted to the granule surface,^42^ roughening its texture,^39,40,43^ but leaving the overall morphology intact.^38,40,44^

The reported effect of cross-linking on the RS content depends on the starch’s botanical source and amylose content. Shin and colleagues found that cross-linking potato, high-amylose corn, and wheat starches results in higher RS content than cross-linking regular corn and rice starches, but precise values depend on the RS measurement method used.^45^ In agreement, cross-linking wheat and high-amylose corn starches has been independently shown to increase RS content more than cross-linked regular corn starch.^39,40,46^

Other starch modifications include hydroxypropylation (reviewed in ref. 47),^47^ acetylation (reviewed in ref. 48),^48^ and octenyl succinylation.^49^ While these modifications increase resistance to varying degrees,^50^ they are generally intended to alter rheological properties,^42^ or encapsulate drugs for delivery to the colon.^49^ Furthermore, USA federal regulations require that esterification with these agents in food products do not exceed 0.1% to 2.5%,^51^ indicating an upper limit to the resistance of chemically modified starches in commercial products.

Some starch modifications occurring during food processing or found in nature can affect its digestibility. Proteins can fortify food matrices,^52^ cell-derived lipids can form complexes at the granule surface,^53^ or glucose molecules can be phosphorylated during glycan synthesis.^54^ In fact, around 0.5% of native potato starch glucose residues are phosphorylated,^54^ up to 5.5 times that of rice starch.^55^ These phosphate monoesters might inhibit exo-acting hydrolases,^46^ or sterically hinder helix packing and reduce overall crystallinity,^56^ particularly during retrogradation.^32^

***Summary***: The available lines of evidence converge to suggest that a starch’s surface microstructure is the principal factor affecting its digestibility. Relative amylose content, amylopectin branch chain density, and crystallinity appear to influence the size, type, and packing density of blocklets, which then determine granule surface texture and porosity. Retrogradation and cross-linking modify starch surface crystallinity and intermolecular networks, respectively, thereby increasing resistance to hydrolysis. It remains unclear whether blocklets simply affect surface area and integrity, or constitute “discrete structures”^57^ that complement amylase active sites. Moreover, these questions tie into whether different bacteria preferentially degrade certain starches more than others based on binding site availability or recognition of discrete microstructures.

## Section 2: Resistant starch degradation by microbes

RS is degraded by the colon’s complex ecosystem of microbes, triggering a cascading web of metabolic interactions. **Primary degraders** grow on RS in monoculture. They penetrate the outer surfaces of intact RS granules, exposing pores and deeper concentric matrices while liberating oligosaccharides and generating metabolites like lactate and acetate.^58,59^ **Secondary degraders** grow on starch in monoculture, but degrade intact RS poorly or not at all. Instead, they may adhere to abrasions and pores on RS before participating in its degradation, and opportunistically utilize solubilized oligosaccharides produced by other RS degraders. **Cross-feeders** do not grow on starch in monoculture. They utilize the by-products generated by upstream degraders, helping to maintain stoichiometric equilibrium and thermodynamically favorable (i.e. unconstrained) fermentation.^60^ Most of the metabolites generated are acidic, which may further stabilize the ecosystem.^58,61^ Together, the subsystem of microbes involved in RS degradation and fermentation participates in a complex network of cross-feeding interactions.^62^ In maintaining microbiome homeostasis, the RS nutrient web expands the scope of what could be considered a “beneficial” gut microbe to a cluster of metabolically interconnected microbes.

Caveats with this model’s classification scheme can be raised. Baxter and colleagues consider secondary degraders and cross-feeders (described below) as butyrogenic “secondary fermenters”.^63^ Likewise, Cerquiera and colleagues define non-primary degrader, starch-active bacteria as “secondary starch scavengers”.^9^ We advance that secondary degraders can be delineated by their ability to grow on starch in monoculture, while a tertiary category (cross-feeders) comprises starch-inactive microbes that play a unique and important role in transforming upstream metabolites. Lastly, some secondary degraders show a limited ability to degrade RS *in vitro*,^64,65^ or toggle their starch activity in the presence of fitter degraders.^66^ Therefore, the “secondary degrader” designation is a fluid concept, but nonetheless useful for the purpose of characterizing individualized responses to RS.

### Unlocking starch: Molecular machinery

Microbial degradation of RS is initiated at the granule surface, which primary and secondary degraders must first recognize and adhere to. Generally, these bacteria possess polysaccharide utilization loci (PULs) encoding transport proteins and modular Carbohydrate-Active Enzymes (CAZymes).^67^ CAZymes contain recognition and catalytic domains.^68^ Endo-acting α-amylases, which hydrolyze internal α-1,4-glycosidic bonds within starch molecules, primarily belong to the family of 13 glycoside hydrolases (GH13).^69^ Other GH families include exo-acting α-glucosidases and the starch debranching enzymes: limit dextrinase, pullulanase, and isoamylase (reviewed in ref. 70).^70^ Alas, most microbial GHs have not been studied in the context of the human gut.^70,71^ This has left a deficit in our understanding of precise GH ligand specificities in relation to RS microstructures.

Starch-active GHs possess one or more carbohydrate-binding modules (CBMs), which are contiguous protein domains that facilitate substrate-specific enzyme docking and enhance catalytic efficiency (reviewed in refs. 69 and 72).^69,72^ Eighty-eight CBM families have been characterized based on amino acid sequence similarity, of which 15 possess starch-binding activities (CBMs 20, 21, 25, 26, 34, 41, 45, 48, 53, 58, 68, 69, 74, 82, and 83).^73^ Early protein crystallization studies showed that CBMs 20, 25, 26, and 34 each recognize glucose residues exposed in starch helical structures.^73^ CBM20 possesses two separate starch surface binding sites with higher specificity for parallel amylose helices than amorphous coils, and an ability to disrupt the structure of shorter helices.^69,74^ Amylases possessing repeated homogenous (e.g. two CBM25 copies)^75,76^ or heterogeneous (e.g. CBMs 25 and 26)^77^ CBMs confer higher avidity and thus tighter adsorption to corn starch granules than those with single CBMs. Of note, most studies investigating CBM ligands use cyclodextrins to infer potential α-glucan helix binding, rather than RS.

Some CAZymes show preferences for starches of different botanical sources.^78–80^ For instance, CBM74 enhances surface pore formation by GHs with higher binding affinity to potato starch granules than wheat and waxy corn.^78^ MaAmyA, a GH13 produced by *Microbacterium aurum* B8.A, uses duplicate CBM25 domains to form pores on wheat, but not potato starch granules.^79^ AmyP, a GH13 found in marine bacteria, exhibits 10-fold higher hydrolytic activity on raw rice compared to potato and wheat starches.^80^ This remarkable difference may simply be due to rice starch’s higher intrinsic digestibility,^26^ but may also be explained by specificity imparted by its starch-binding domain, CBM69.^81^ At present, there is no obvious pattern by which the copy number of GH13’s or CBM family domains can predict whether a bacterial strain can degrade starch or RS (Table 2).

### Primary degraders

By using complex enzymatic machinery to dock and digest starch granules, certain RS-degrading specialists find a niche in “unlocking” RS granules for other RS guild members to access and ferment. *Ruminococcus bromii* and *Bifidobacterium adolescentis* are the best characterized primary degraders thus far.

#### Ruminococcus bromii

*R. bromii* is considered a keystone species in the colon,^65^ comprising 3% of fecal bacteria in Europeans.^82^ *R. bromii* uniquely possesses an amylosome – a multiprotein complex composed of extracellular GHs flanked by additional CBMs (including CBM26 and 48) that associate via calcium-dependent cohesion–dockerin interactions.^83^ Across five different *R. bromii* strains, there are 17 conserved GH13-containing amylases (Amy1-17) that enable hydrolysis of both α-1,4- and α-1,6-glycosidic bonds.^84,85^ In addition to liberating more starch by-products (i.e. malto-oligosaccharides, maltose, and glucose) than it requires to grow,^65^ *R. bromii* generates acetate, ethanol, formate, and propanol.^59^ Unlike other notable Ruminococcaceae family members, *R. bromii* has not been shown to generate butyrate.^59,^^65,,86^

*In vitro, R. bromii* strains L2-63, L2-36, 5AMG, YE282, and ATCC 27255 show amylolytic activity on retrograded (Novelose 330) and high-amylose (Hi-Maize 958) corn starches.^84^ In strain L2-63, these activities are higher than those on potato starch.^83^ Intriguingly, *R. bromii* L2-63 grows better on native potato starch than on cross-linked potato starch (Versafibe 1490), while it grows better on cross-linked high amylose corn starch (HACS; Versafibe 2470) than on native HACS.^64^ Considering that the integrity of native starch reaching the colon depends on host factors, these *in vitro* observations may not reflect *R. bromii’s* substrate preference in the colon. This is supported by *R. bromii’*s response to RS *in vivo*, contradicting its preferences seen *in vitro* (discussed in Section 3).

#### Bifidobacteria adolescentis

*Bifidobacteria* possess a multi-modular carbohydrate-utilization system (reviewed in ref. 9)^9^ enriched with GHs with specificity for a broad range of glycan substrates.^87^ CBMs 25, 26, and 74 are thought to work synergistically to enable *Bifidobacteria* to not only dock starch,^9^ but also agglutinate starch granules into clusters.^85^ This behavior is thought to sequester granules away from competing starch-degraders.^88^ Within the *Bifidobacteria* genus, only *B. pseudocatenulatum* strains M115 and DSM 20438,^58,89^ and *B. adolescentis* have been shown to digest RS in humans.^65^ *B. adolescentis* comprises 0.25% to 1.4% of the fecal microbiota in European individuals.^90,91^

Fifteen strains of *B. adolescentis* have been isolated from humans.^64,92–94^ Of the strains whose activity on starch has been studied *in vitro*, two (P2P3 and L2-32)^65,93^ utilize RS, while four do not (703B,^92^ DSM 20083, DSM 20086,^93^ and NCFB 2229).^95^ Nonetheless, strains that can ferment RS generate lactate, acetate, and formate.^58^ *B. adolescentis* uses more of its starch degradation by-products than *R. bromii*, including glucose, signaling a more competitive relationship with other RS guild members.^65^ Intriguingly, Li and colleagues found that an unspecified strain of *B. adolescentis* produces butyrate,^96^ in contrast with prior reports that the species is not butyrogenic.^58^

*B. adolescentis* shows strain-specific preferences for different RS. Strain P2P3 utilizes HACS (Hi-Maize 958) to a significantly higher degree than cross-linked corn starch (Versafibe 2470), cross-linked potato starch (Versafibe 1490), retrograded corn starch (Novelose 330), and most intriguingly, a different HACS (Hi-Maize 260).^93^ Strain VTT E-001561 exhibits preferential binding (i.e. adherent cell counts normalized per gram of starch) to HACS (Hylon VII), and lowest binding to potato starch.^94^ These results require careful interpretation because potato starch granules have smaller surface area-to-volume ratios (and thus less binding area) than corn starch. Furthermore, strain IVS-1 shows preferential growth on HACS (Hi-Maize 260), lesser but considerable growth on native potato starch and cross-linked tapioca starch (Versafibe 3490), and little growth on cross-linked corn (Versafibe 2470) and potato (Versafibe 1490) starches.^64^ The putatively butyrogenic *B. adolescentis* strain described earlier will adhere to and grow better on partially hydrolyzed than non-hydrolyzed retrograded tuber starch granules, likely because of its rougher yet more crystalline surface.^96^ Together, these studies seem to indicate *B. adolescentis* has a nuanced preference for HACS over other types of RS *in vitro*; however, this finding has not yet been recapitulated by *in vivo* microbiome studies.

*B. choerinum* FMB-1, isolated from bovine rumen fluid, degrades cross-linked potato starch (Versafibe 1490) and HACS (Hi-Maize 958), but exerts negligible activity on regular (Hi-Maize 260), retrograded (Novelose 330), and cross-linked HACS (Versafibe 2470) starches.^93^ *B. pseudocatenulatum* strain DSM 20026,^97^ but not NCIMB 8811, grows on potato starch alone,^95^ and M115 grows on retrograded corn starch (Novelose 330).^89^ However, Novelose 330 is approximately half digestible starch by weight,^98^ thereby detracting the likelihood that this strain is a true primary degrader.

Primary degraders are thought to be necessary for RS degradation in the human gut. Work by the Flint laboratory revealed that individuals consuming RS whose butyrate-production did not increase (considered to be “RS non-responders”) harbored nearly undetectable levels of *R. bromii* as compared to RS responders.^5^ In a follow-up *in vitro* study, Flint et al. cultured stools from RS non-responders with spiked-in *R. bromii* L2-63, which boosted RS-fermentation to levels comparable to RS responders.^65^ This work was seminal for developing a model whereby primary degraders (e.g. *R. bromii*) “unlock” RS for other community members to degrade and ferment.

### Secondary degraders

*Secondary* degraders possess extracellular amylases to degrade regular starch, but their contribution to initiating RS degradation is negligible compared to that of primary degraders. Instead, they may require primary degraders to erode smooth RS granule surfaces before adhering to RS and/or scavenging for “substrate spillover” (i.e. excess oligosaccharides generated by primary degraders).^58^ Conceivably, it is advantageous for secondary degraders to position themselves near primary degraders docked to RS. Among secondary degraders, preferences for certain starches over others have not been greatly elucidated by metaproteomic, co-culture, or clinical studies.

#### Eubacterium rectale

*Eubacterium rectale* is a prominent member of the butyrate-producing *Clostridium* cluster XIVa, which is implicated in maintaining gut homeostasis.^99^ *E. rectale* L1-86 is equipped with 11 GH13s, of which two have been deeply characterized: Amy13K and Amy13B.^100^ Amy13K contains duplicate CBM26 domains and additional CBM41, 82, and 83 domains, enabling *E. rectale* to bind to certain starch granules (discussed below).^101^ *In vitro*, Amy13K hydrolyzes amylopectin with twice the activity than amylose, releasing maltotetraose and maltopentaose.^100^ These solubilized substrates, liberated either by *E. rectale* or primary degraders, may then be further hydrolyzed by Amy13B, which does not bind larger substrates.^100^ Finally, malto-oligosaccharides of DP less than 8 are transported into the cell via EUR_01830, EUR_31480, and EUR_01240 ABC-transporters.^100^

**Figure 2.**
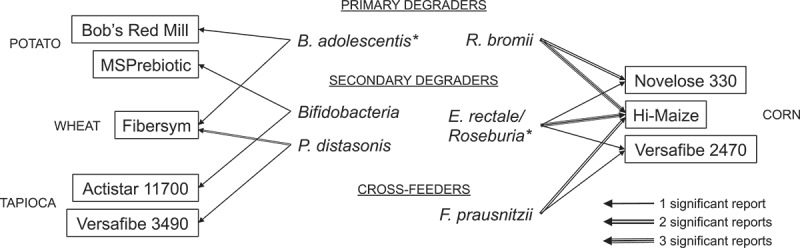
**RS guild members show preferences for different RS *in vivo***. Primary degraders, secondary degraders, and cross-feeders discussed in-text that have been reported to significantly increase in relative abundance across 16 clinical trials where the microbiome was monitored. Lines indicate the frequency of reported associations. *Species-level differences among *B. adolescentis* and *E. rectale/Roseburia* could not be resolved in every study. Full data can be found in **Supplemental Table 1.**

Like primary degraders, *E. rectale* exhibits RS preferences. *E. rectale* DSM 17629 shows detectable growth on cross-linked corn starch (Versafibe 2470), but not cross-linked potato (Versafibe 1490) or tapioca (Versafibe 3490) starches.^64^ Furthermore, it can bind and hydrolyze regular and high-amylose corn starches (Hylon VII,^100,102^ Hi-Maize 260,^64,101^ and Hi-Maize 958),^65^ but binds potato and cross-linked wheat starch (Fibersym) granules less efficiently.^101^ Human fecal inocula cultured in a batch-fermentation system revealed that *E. rectale* responds equally to A-type and B-type retrograded HACS.^35^ In this study, *E. rectale*’s growth lagged behind *Bifidobacteria*, potentially indicating a reliance on primary degraders to erode RS first.^35^ In agreement with its behavior *in vitro, in vivo* microbiome studies show that the relative abundance of *E. rectale* is increased in response to regular (Hi-Maize 260), retrograded (Novelose 330), and cross-linked (Versafibe 2470) HACS (Figure 2).

*E. rectale* enrichment is positively associated with increased butyrate following potato starch intervention.^11^ It interacts synergistically with *R. bromii* in co-culture,^65^ and these species consistently co-enrich in RS intervention studies.^5,15,102,103^ Considering the apparent reliance upon *R. bromii* in initiating starch degradation,^65^ it may ultimately be that any potential preference *E. rectale* (and other secondary degraders) has for different RS is overshadowed by that of its associated primary degrader, which then dictates overall RS fermentation.

#### Roseburia and Butyrivibrio

n addition to *E. rectale*, other starch-degrading Lachnospiraceae include butyrogenic species belonging to the *Roseburia* and *Butyrivibrio* genera. *R. faecis* M72/1 grows on amylopectin,^67,104^ but negligibly on amylose,^67^ and its amylolytic machinery has not been characterized. *R. inulinovorans* A2-194 uses Amy13A, a GH13 containing CBM41, 48, 82, and 83 domains,^101,105^ to grow on amylopectin.^106^ This strain upregulates amylase and flagella expression when provided with amylopectin.^107^ Both *R. inulinivorans* A2-194 and *R. intestinalis* L1-952 and L1-82 grow poorly on HACS compared to more readily digestible rice, corn, and waxy potato and corn starches, and *B. fibrisolvens* 16/4 shows the same general preferences.^108^ *B. fibrisolvens’* Amy13B contains two tandem CBM26 and CBM83 domains,^101^ with higher activity against rice starch than Amy13A from *R. inulinivorans*.^108^ Lastly, *B. crossotus*-related taxa have been found to adhere to HACS (Hylon VII).^102^

#### Bacteroides thetaiotaomicron

*B. thetaiotaomicron* is endowed with 88 different polysaccharide utilization loci (PULs) encoding 226 GHs,^109^ enabling it to utilize at least 32 different types of glycans.^110^ *B. thetaiotaomicron* also possesses an intricate starch-utilization system (Sus). The first PUL to be described,^111^ the *sus* operon encodes independent intracellular (SusR), periplasmic (SusAB), transmembrane (SusC), and cell wall-anchored (SusDEFG) proteins with coordinated action, briefly described in their order of action here (thoroughly reviewed in ref. 112).^112^ SusD is a CBM-analog necessary for growth on starch granules,^113^ and binds the endo-regions of α-glucan helices.^114^ SusG is a GH13 α-amylase that contains CBM58 and a starch surface-binding site, with a twofold higher activity on soluble potato starch than amylopectin and strict hydrolytic-specificity for α-1,4-glycosidic bonds.^115^ SusG cooperates with SusE and F to bind soluble and granular starch.^113^ Eliminating all SusEFG starch-binding sites slows growth on amylopectin derived from corn more than potato.^113^ While not required for cell growth,^116^ SusE assists in capturing DP 7–18 malto-oligosaccharides,^117^ and both SusE and SusF bind wound and unwound helices.^116^ SusC contributes to starch binding and imports malto-oligosaccharides into the cell,^118^ which are degraded into maltose and glucose by periplasmic SusA and B.^111^ Lastly, constitutively expressed SusR is activated by intracellular maltose to rapidly upregulate *sus* gene expression.^119^

*B. thetaiotaomicron* generates lactate, acetate, and propionate, and while it does not produce butyrate,^120^ its metabolites drive butyrate production by other bacteria via cross-feeding interactions.^121^ Most of *B. thetaiotaomicron*’s GHs localize to the periplasm or outer membrane, thereby contributing to the soluble glycan pool for community use.^122^ *B. thetaiotaomicron* may also use SusE and SusF to sequester starch from its competitors.^116^ Furthermore, when *B. thetaiotaomicron* and *E. rectale* co-colonize gnotobiotic mice, *B. thetaiotaomicron* upregulates its starch and host-glycan degradation pathways, while *E. rectale* downregulates its GH expression and increases expression of transport proteins, relying instead on butyrate-producing pathways to generate energy.^66^ Interestingly, when provided both pectic galactan and amylopectin, *B. thetaiotaomicron* will downregulate Sus expression and upregulate expression of other PULs, indicating a substrate preference for non-starch polysaccharides.^123^ Indeed, starch availability and interspecies competition may relegate secondary degraders to cross-feeders, or vice versa.

*B. thetaiotaomicron* can utilize less than 20% of raw (Hi-Maize 958) and retrograded (Novelose 330) HACS after a 72-hour incubation, but cannot utilize a different type of HACS (Hi-Maize 240).^65^ However, *B. thetaiotaomicron* grows with these starches better if co-cultured with *R. bromii* or *B. adolescentis*, or if the starches are autoclaved or boiled.^65^ Hence, while *B. thetaiotaomicron* prefers other glycans, it is equipped with a sophisticated system to access starch, and can also influence the activity of other RS guild members.

#### Bifidobacteria

Several *Bifidobacteria* beyond those mentioned above show strain-specific potential to be secondary degraders (Table 1). These species include *B. longum, B. bifidum, B. breve, B. dentium, B. infantis, B. pseudolongum, B. thermophilum*, and *B. angulatum. B. breve* produces ApuB, a GH13 amylase with CBMs 25, 41, and 48 domains.^124^ ApuB cleaves both α-1,4- and α-1,6-glycosidic bonds using different active sites, and is required by *B. breve* UCC2003 to grow on starch.^125^ *B. breve* 20213 shows similar growth patterns as *B. adolescentis* L2-32 on waxy corn, high-amylose corn, wheat, and rice starches,^65^ and an unspecified strain of *B. breve* has been shown to bind HACS (Hylon VII).^102^

**Table 1.** **Strain-specific differences in starch utilization**. Taxa that have demonstrated utilization, growth, or binding to starch. References supporting each observation can be found in **Supplemental Table 2.**

Species	Degrader	Starch Non-Degrader
*R. bromii*	L2-63	
	L2-36	
	5AMG	
	YE282	
	ATCC 27255	
*B. adolescentis*	P2P3	703B
	22 L	DSM 20083
	L2-32	DSM 20086
	DSM 24849	DSM 20086
	VTT E-001561	NCFB 2229
	CSCC 5305	
	IVS-1	
	CIP 64.60	
	CIP 64.61	
*B. choerinum*	FMB-1	
*B. pseudocatenulatum*	M115	NCIMB 8811
	DSM 20438	IPLA 20026
*E. rectale*	A1-86	
	DSM 17629	
*R. inulinovorans*	A2-194	
*R. intestinalis*	L-952	
	L1-82	
*B. fibrisolvens*	16/4	
*P. distasonis*	ATCC 8503	
*B. thetaiotaomicron*	VPI-5482	
	5482	
*B. longum*	JCM 7050	JCM 7052
	CIP 64.63	JCM 7053
		JCM 7055
		JCM 7056
		CCUG 15137
		CCUG 30698
		IPLA 20027
*B. longum* subsp. *longum*	NCIMB 8809	BBMN68
		NCIMB 8809
*B. bifidum*	70/18	JCM 7002
	VTT E-001559	JCM 7003
		NCIMB 8810
		CIP 64.65
		CCUG 17358
		IPLA 20015
*B. breve*	UCC 2003	DSM 20006
	JCM 7019	DSM 20213
	CCUG 43878	
	CCUG 34405	
	NCFB 2258	
	ATCC 20213	
*B. dentium*	NCFB 2243	
*B. globusum*	JCM 5820	
	JCM 7092	
*B. longum* subsp. *infantis*	CCUG 45868	20088
*B. pseudolongum*	NCIMB 2244	
	DSM 20095	
	ATC 25526	
*B. pseudolongum* subsp. *globosum*	DSM 20092	
*B. thermophilum*	JCM 7027	
*B. angulatum*	ATCC 27535	
	DSM 20098	
*B. infantis*	CCUG 45868	CCUG 36569
		NCDO 2205

**Table 2.** **Investigating starch utilization, and GH13 and CBM abundances**. The number of GH13’s and starch-binding CBMs encoded by select bacteria discussed in text, with available genomes obtained from the CAZyme database (www.cazy.org).^149^ Symbols indicate whether strains can (+), weakly (o), or cannot (-) degrade starch or resistant starch (RS). Of note, five starch-binding CBM families have not been observed in the listed genomes (CBM 21, 45, 53, 68, and 69)

Species	Strain	Starch	RS	GH13	CBM20	CBM25	CBM26	CBM34	CBM41	CBM48	CBM58	CBM74	CBM82	CBM83
*R. bromii*	L2-63	+	+	15			3			6		1		
*B. adolescentis*	P2P3	+	+	17		5	4		2	5		1		
*B. adolescentis*	22 L	+	+	16		4	4		2	4		1		
*B. choerinum*	FMB-1	+	+	14		3	1		2	4		1		
*E. rectale*	DSM 17629	+	o	13			2	1	1	4			1	1
*R. intestinalis*	L1-82	+	-	13				1		4			2	
*B. fibrisolvens*	16/4	+	-	10			3	2		1				
*P. distasonis*	ATCC 8503	+	o	7	2					1				
*B. thetaiotaomicron*	VPI-5482	+	o	8	2						1			
*B. breve*	UCC 2003	+	-	14		1			2	4				
*B. breve*	JCM 7019	+	-	12						4				
*B. breve*	NCFB 2258	+	-	13		1			2	4				
*B. pseudolongum*	DSM 20092	+	-	17		5	1		2	5		1		
*B. angulatum*	DSM 20098	+	-	13	4		3		1	5		1		
*B. longum*	BBMN68	-	-	13						3				
*B. longum*	DSM 20088	-	-	8						3				
*B. longum*	CCUG 30698	-	-	12						3				
*B. breve*	DSM 20213	-	-	12		1			2	4				

*B. cuniculi* and *B. magnum* both grow better on starch when co-cultured than in monocultures.^87^ In contrast, only *B. thermacidophilum* subsp. *porcinum*’s growth on starch is improved when co-cultured with *B. longum* subsp. *suis*. Indeed, *B. longum* subsp. *suis* growth is unchanged, suggesting *porcinum* outsources starch degradation to *suis*.^87^ While these two pairs of *Bifidobacteria* were isolated from rabbit and pig feces, respectively, they serve to illustrate that relationships between secondary degraders range from mutualistic to commensal.

Other potential starch-degraders have been identified but studied less extensively than those listed above. *Lactobacillus amylovorus* uses AmyA, an α-amylase containing five tandem CBM26 repeats, to bind to raw corn starch.^76,126^ *Parabacteroides distasonis* ATCC 8503 grows on native and cross-linked corn (Versafibe 2470) and potato (Versafibe 1490) starches in culture without a distinct preference, but achieves stationary phase after 4 days rather than 12 to 24 hours as seen with *B. adolescentis* and *R. bromii*.^64^ Two uncharacterized species belonging to Ruminococcaceae and Clostridiaceae have been inferred to be RS degraders that are markedly enriched in subjects consuming either HACS (Hi-Maize 260) or potato starch.^63^ Lastly, a sequenced taxon most closely related to *Ruminoclostridium* [*Eubacterium*] *siraeum* increased in relative abundance in one subject fed potato starch whose *R. bromii* and *B. adolescentis* did not enrich.^11^

### Cross-feeders

RS cross-feeders utilize starch by-products or metabolites generated by upstream RS degraders, such as acetate, lactate, formate, and succinate. *R. hominis* A2-183 does not grow on starch in monoculture, but it does grow in co-culture with *B. adolescentis* L2-32, by utilizing substrate spillover and/or the acetate and lactate produced by *B. adolescentis*.^58^ Furthermore, *R. hominis* A2-183 is unable to degrade amylopectin or amylose,^67^ suggesting that it degrades smaller malto-oligosaccharide fractions. The *R. hominis* genome encodes fewer GH13s (8) than *R. intestinalis* (12 to 13) and *R. inulinivorans* (10 to 12), and it does not encode GH13s possessing CBM26 or CBM41 domains.^67^ Considering the high prevalence of these CBMs across known starch degraders (Table 2), their absence may account for *R. hominis*’ limited capacity to utilize starch-derived molecules, and would therefore be better considered a cross-feeder than secondary-degrader.^67^ Similarly, *R. gnavus* ATCC 29149 cannot grow on soluble or retrograded starch alone, but cross-feeds upon substrate spillover when grown in co-culture with *R. bromii*.^59^

Describing all known gut bacteria capable of utilizing these substrates exceeds the scope of this review, but one other example is noteworthy. *F. prausnitzii* is a prominent butyrate-producing commensal, comprising 1.5% to 9.5% of fecal bacteria in European individuals.^127^ *F. prausnitzii* utilizes maltose and acetate to generate butyrate.^86,128^ Among 10 *F. prausnitzii* strains, growth on starch and amylopectin is negligible or undetectable in monoculture.^97,104,129^ Other butyrate-producing bacteria found in the colon are reviewed in ref. 130.^130^ Of note, *F. prausnitzii* (and primary and secondary degraders) are depleted in health conditions associated with low butyrate production, like inflammatory bowel disease.^131,132^

## Section 3: *In vitro* and *In vivo* microbiome studies

Our nascent understanding of how individual bacteria respond to different RS can be complemented by monitoring their activity in the full ecosystem of the gut microbiome. Two common approaches are fermentation (*in vitro*) and intervention (*in vivo*) studies. Batch fermentations involve inoculating fecal material into a pH-, temperature-, and anaerobically maintained system that attempts to replicate the gut microenvironment.^40,133–135^ By administering RS to these cultures, temporal changes occurring in microbial populations can be readily monitored. While used less frequently, continuous flow fermentations involve cycling gases and substrates in and out of the system to further maintain physiologically relevant conditions.^35,136^

RS intervention studies generally involve double-blinded clinical trials, whereby subjects ingest RS and/or non-RS placebo for several weeks. In parallel-armed trials, subjects are randomly divided into RS and control groups. However, inter-individual variation is better controlled with cross-over designs, whereby subjects alternate between RS and control groups, often punctuated by 2-week washout periods to restore the microbiota toward baseline. Fecal samples are collected before, during, and after the intervention to analyze bacterial, metabolite, and protein abundances. Although informative in their own right, animal models will not be discussed here because of uncertainties introduced by host factors (e.g. oro-cecal transit time, amylase activity, and hindgut fermentation) that have not been robustly shown to recapitulate human physiology.

### In vitro microbiome studies

The advantages of *in vitro* microbiome studies include eliminating upstream host variables, predicting bacterial responses in different biogeographical regions of the colon by tweaking media pH, and monitoring changes in bacterial populations over time. For example, Lesmes and colleagues devised a three-compartment continuous-flow fermentation system with pH ranges corresponding to the proximal (pH 5.5), transverse (pH 6.2), and distal (pH 7.1) colon.^35^ Using stools obtained from three healthy volunteers, they found that *Bifidobacteria* grew equally well with B-type crystallite-enriched HACS across all pH ranges, but grew most poorly with A-type HACS at pH 5.5. Growth curves indicated that total bacteria declined over the first 10 hours of culture, followed by a compensatory climb for 14 hours, and that *E. rectale*’s growth lagged 5 hours behind that of *Bifidobacteria* when grown with HACS, consistent with their putative roles in the RS guild. Of note, B-type HACS induced a fivefold higher increase in butyrate compared to A-type after 24 hours of culture.

By incubating stool with HACS cross-linked under different concentrations of STPP/STMP, Wang and colleagues showed that *R. bromii* enrichment is inversely proportional to the degree of RS cross-linking.^40^ Regular HACS significantly enriches *R. bromii* after 4 hours followed by a depletion in the species after 12 hours of incubation, whereas HACS cross-linked in a 12% w/w STMP/STPP solution yields a less pronounced but stable enrichment over 4–24 hours. This work also indicated that RS can potentially become too resistant to hydrolysis even for microbes specialized to degrade it. Furthermore, enrichment of *R. faecis* and an unclassified *Clostridiales* member lagged behind *R. bromii*, parallel to the relationship seen between *Bifidobacteria* and *E. rectale* in Lesmes and colleagues’ work.^35^

Li and colleagues introduced a novel high-throughput stool fermentation assay, called *RapidAIM*,^134^ which they employed to assess type 2, 3, and 4 RS’ in stool slurries collected from six healthy volunteers.^135^ While no effect was seen in response to cross-linked wheat starch, regular HACS (Hi-Maize 260) and retrograded HACS (Novelose 330) enriched *E. rectale, R. faecis, Roseburia*, and *Lachnospiraceae*. The retrograded HACS also enriched *Bifidobacterium, Subdoligranulum variabile*, and *Ruminococcaceae*.

### In vivo microbiome studies

One rationale behind clinical trials involving RS is to selectively enrich butyrate-producing bacteria and increase butyrate production. To our knowledge, 16 studies have reported changes in the fecal microbiome of individuals following RS interventions (**Supplemental Table 1**). While acknowledging that these studies’ methodologies and experimental designs differ, patterns have emerged to show that RS guild members respond to RS *in vivo* according to RS type (Figure 2). Together, these studies underscore the nuanced responses of previously identified primary and secondary degraders, and cross-feeders to RS ingested over several weeks. Beyond these observations, they also lend insight into bacterial co-associations, butyrate production, and inter-individual variability.

#### Bacteria co-associations

Among individuals supplemented with potato starch or HACS (Hi-Maize 260), Baxter and colleagues identified several co-associations between primary degraders and butyrogenic taxa. Hi-Maize 260 significantly enriched *R. bromii*, which was significantly associated with co-enrichments of *E. rectale*. Likewise, potato starch significantly enriched the *B. faecale/adolescentis/stercoris* group, which was significantly associated with co-enrichments of *Anaerostipes hadrus*.^63^ *A. hadrus* has previously been shown to grow on starch in co-culture with *R. bromii*, but not in monoculture.^137^

After 12 weeks of a cross-linked wheat starch (Fibersym) intervention, positive co-associations between 18 pairs of bacteria were observed, including co-enrichments of *Bacteroides acidifaciens* and *Bacteroides ovatus*, and *Christensenella minuta* and *Ruminococcus lactaris*.^138^ Of note, *F. prausnitzii* did not correlate with any of these species, yet butyrate production was significantly increased following RS intervention, indicating potentially novel butyrogenic RS guild members.

Deehan and colleagues identified 55 taxa that were affected by dose-dependent cross-linked RS intake, which they further categorized into seven distinct co-abundance response groups (CARGs).^64^ These co-abundance response groups (CARGs), containing 3 to 18 taxa each, were constructed by hierarchical clustering of Spearman’s correlation distances. Then, each CARGs’ constitutive taxa were summed together into a single entity for downstream statistical analyses. They found significant correlations among CARG1 (including *B. adolescentis, P. distasonis, Eubacterium hallii*, and others) and CARG7 (including several *Eubacterium and Bacteroides* species) with cross-linked tapioca starch (Versafibe 3490), and CARG3 (including *Ruminococcus callidus* and *Bacteroides plebeius*) with cross-linked corn starch (Versafibe 2470).^64^ Employing CARG-based analyses in this way enables the detection of underlying ecological guilds, boosting otherwise weak signals of individual taxa. Guild-based approaches have been proposed by others.^139^ Interestingly, Deehan et al. also reported a significant co-exclusion between *R. bromii* and an unannotated *Ruminococcus* taxon (OTU27), suggesting a potential novel primary degrader that competes for *R. bromii’s* niche.

#### Butyrate production

Butyrate is a fermentation end-product known for its immunomodulatory bioactivities.^2^ Among the 16 studies listed in **Supplemental Table 1**, seven reported the butyrate concentrations in fecal samples, and potato starch consistently leads to significant increases in butyrate concentrations.^3,11,63^ Cross-linked wheat starch (Fibersym) was also found to significantly increase fecal butyrate.^138^ In contrast, various HACS led to no significant changes between groups.^63,140,141^ Cross-linked HACS (Versafibe 2470) led to significant increases in butyrate concentrations, plateauing at a dose of 35 g RS per day, while no significant changes were seen with any dose of cross-linked potato (Versafibe 1490) or tapioca (Versafibe 2490) starches.^64^ In the cross-linked HACS (Versafibe 2470) group of this study, *R. bromii* was significantly negatively associated with the proportion of butyrate relative to acetate and propionate, yet there was a significant positive association in the cross-linked potato starch group. Lastly, retrograded HACS (Novelose 330) significantly *decreased* butyrate in fecal samples from British males.^142^ Since it is estimated that 95% of short-chain fatty acids are absorbed by the host epithelium, fecal butyrate concentration has been implicated as a low-fidelity proxy for intestinal butyrate production.^143^ Thus, the discrepant and counter-intuitive changes in butyrate concentrations may be explained by methodological limitations, or nuanced RS guild dynamics and underlying effects seen at the individual, but not group level, described below.

#### Inter-individual variability

Several *in vivo* studies report bacterial responses for each subject, showcasing how *R. bromii, B. adolescentis*, and *E. rectale*, may increase, remain unchanged, or even decrease in response to different RS depending on the individual.^4,64,103,142,144^ Based on the *in vitro* behavior of RS guild members, we would expect to see these taxa more consistently enriched following RS interventions. This discrepancy may be explained by biological factors and methodological limitations.

Biological factors that may explain inter-individual variability include the presence/absence of keystone RS guild members,^65^ functional subgroup variability,^145^ metabolic flux sensitivities,^136^ and baseline diet variability.^10^ First, Ze and colleagues demonstrated that *R. bromii* can be necessary for RS degradation *in vivo*.^65^ Second, if keystone RS degraders are present in an individual, they may lack downstream subgroups required for converting RS into butyrate.^145^ For instance, possessing lactate-producing *B. adolescentis*, but lacking lactate-utilizing butyrogens (e.g. *E. hallii*) may result in a poor butyrate response to RS; likewise, if one possesses net acetate-producing *R. bromii* but lacks acetate-utilizing butyrogens (e.g. *F. prausnitzii)*. Third, the dominating metabolites produced by the RS guild may exert feedback on the global microbiome structure. For instance, without sufficient microbial lactate utilization, lactate produced by *B. adolescentis* may accumulate, lower luminal pH,^59^ stress pH-sensitive microbes, and destabilize the microbiome.^136^ So far, these insights point to the potential utility of an RS guild member probiotic, synbiotic, or consortium which may enable RS non-responders to benefit from RS therapies. Lastly, McOrist and colleagues observed that individuals with the highest baseline butyrate production tended to decrease butyrate production following a dietary intervention high in RS.^10^ They commented that these individuals’ pre-treatment diets and microbiomes were likely configured optimally to generate butyrate, and exchanging their dietary patterns for the intervention diet may have disrupted their butyrogenic system. These biological factors underscore that interventions ought to be personalized where butyrate-production is a primary endpoint.

Methodological limitations that may contribute to inter-individual variability include poor taxonomic resolution, underpowered sample sizes, and compositionally insensitive statistical techniques. First, 16S amplicon sequencing and genus-level qPCR primers used in several of these studies were unable to resolve and differentiate RS- and starch-degrading species and strains (e.g. *R. bromii* and *B. adolescentis*) from their starch-inactive phylogenetic relatives (Table 1).^5,7,15^ Second, many studies may not have been sufficiently powered to draw robust conclusions about species-level changes, with sample sizes ranging between 8 and 46 subjects per treatment group. With larger sample sizes, subgroup analyses like those employed by Venkataraman et al. may boost the detection of otherwise weak signals at the study population level. For instance, k-means clustering of participants based on changes in fecal butyrate concentration revealed that *B. adolescentis* and *R. bromii* significantly enrich in RS responders, and *E. rectale* significantly enriches in individuals with high butyrate production at baseline.^11^ Lastly, microbiome sequencing data are compositional, meaning that gene amplicon read counts do not necessarily reflect bacterial absolute abundances.^146^ Instead, read counts are typically normalized to sum to 100%. For this reason, the relative abundances of smaller keystone communities (e.g. primary degraders) may increase, but appear to decrease simply because cross-feeders increase in relative abundance to a greater extent.^146^ While compositionally sensitive techniques like the centered log ratio transformation can help mitigate this technical artifact,^146^ microbiome responses can also be evaluated by monitoring beta-diversity (i.e. overall microbial community composition). Plotting fecal microbiota compositions in two-dimensional space reveals multidirectional changes across individuals before and after RS interventions, signaling that changes are driven by different taxa communities.^7,144^ Together, these limitations illustrate the necessity of sufficiently powering RS interventions where microbiome composition is the primary endpoint, collecting critical baseline data and employing appropriate statistical techniques.

## Conclusion

RS-based interventions have been proposed as a safe and economical approach to restoring gut microbiome homeostasis through the selective enrichment of butyrate-producing bacterial communities. However, there are several commercial RS products available, each with different physicochemical properties affecting their hydrolysis by endogenous and bacterial amylases. Within the microbiome, we see that microbes show preferences for different types of RS, which is made clearer by categorizing primary degraders, secondary degraders, and cross-feeders based on their ability to grow on RS and regular starch. We advance that the involvement of starch-inactive cross-feeders is critical to the net metabolic outcome of RS interventions. Indeed, how the microbiome composition and function changes is dependent on microbe-, host-, and RS-specific factors.

At present, there are several knowledge deficits surrounding these factors, which once elucidated, may enable data-driven RS-selection based on individual microbiome features:

- What determines whether RS guild members are co-exclusive or co-abundant? Multiple species likely compete for the same role in the RS guild, but generate different metabolites that affect the composition of downstream members. For instance, the co-exclusive primary degraders *R. bromii* and *B. adolescentis* predominately produce acetate and lactate, respectively, which likely favors guild membership to acetate- or lactate-consuming secondary degraders and cross-feeders.
- What determines which type of RS bacteria will bind and degrade? Do bacterial CAZymes recognize discrete microstructures on starch granules (thus conferring substrate selectivity), are certain combinations of CAZymes necessary for effective penetration of RS, or does metabolic feedback select for certain RS guild members over others?
- To what degree do host factors (e.g. amylase gene copy number) influence RS digestion prior to entry into the colon? Incorporating these data may improve RS-selection for individuals. Habitual dietary RS intake may also contribute to variable responses,^147^ but dietary records are rarely collected.
- How do other kingdoms (e.g. fungi) contribute to RS degradation in the gut?
- Lastly, butyrate production is not the sole indicator of microbiome functionality. Can RS restore gut dysbiosis in other ways, such as by regulating microbial bile acid or tryptophan metabolism?^148^

## Supplementary Material

Supplemental Material
