# Supplementary material for: Resistant starch, microbiome, and precision modulation
Source: Gut Microbes. 2021 Jul 18;13(1):1926842. doi: 10.1080/19490976.2021.1926842 (PMC8288039; doi:10.1080/19490976.2021.1926842)
Supplement: Supplemental Material [file KGMI_A_1926842_SM8486.zip › Supplementary information/Supplemental Tables caption.docx]

**Supplemental Table 1. 16 clinical trial studies published between 2004 and 2020 where subject microbiome compositions were analyzed.**

# Supplemental Table 2. Expanded Table 1 highlighting the utilization, growth, and binding of starch-degrading bacteria.
